# Acupuncture on mild cognitive impairment: A systematic review of neuroimaging studies

**DOI:** 10.3389/fnagi.2023.1007436

**Published:** 2023-02-15

**Authors:** Zihan Yin, Jun Zhou, Manze Xia, Zhenghong Chen, Yaqin Li, Xinyue Zhang, Xiang Li, Hang Yan, Lu Wang, Mingsheng Sun, Ling Zhao, Fanrong Liang, Ziwen Wang

**Affiliations:** ^1^School of Acu-Mox and Tuina, Chengdu University of Traditional Chinese Medicine, Chengdu, China; ^2^Acupuncture Clinical Research Center of Sichuan Province, Chengdu, China; ^3^School of Basic Medicine, Zhejiang University of Traditional Chinese Medicine, Hangzhou, China

**Keywords:** acupuncture, mild cognitive impairment, systematic review, neuroimaging, mechanism

## Abstract

Mild cognitive impairment (MCI) is a multifactorial and complex central neurodegenerative disease. Acupuncture appears to be an effective method for cognitive function improvement in MCI patients. Neural plasticity remaining in the MCI brain implies that acupuncture-associated benefits may not be limited to the cognitive function. Instead, neurological alternations in the brain play a vital role in corresponding to the cognitive improvement. However, previous studies have mainly focused on the effects of cognitive function, leaving neurological findings relatively unclear. This systematic review summarized existing studies that used various brain imaging techniques to explore the neurological effect regarding acupuncture use for MCI treatment. Potential neuroimaging trials were searched, collected, and identified independently by two researchers. Four Chinese databases, four English databases, and additional sources were searched to identify studies reporting the use of acupuncture for MCI from the inception of databases until 1 June 2022. Methodological quality was appraised using the Cochrane risk-of-bias tool. In addition, general, methodological, and brain neuroimaging information was extracted and summarized to investigate the potential neural mechanisms by which acupuncture affects patients with MCI. In total, 22 studies involving 647 participants were included. The methodological quality of the included studies was moderate to high. The methods used included functional magnetic resonance imaging, diffusion tensor imaging, functional near-infrared spectroscopy, and magnetic resonance spectroscopy. Acupuncture-induced brain alterations observed in those patients with MCI tended to be observable in the cingulate cortex, prefrontal cortex, and hippocampus. The effect of acupuncture on MCI may play a role in regulating the default mode network, central executive network, and salience network. Based on these studies, researchers could extend the recent research focus from the cognitive domain to the neurological level. Future researches should develop additional relevant, well-designed, high-quality, and multimodal neuroimaging researches to detect the effects of acupuncture on the brains of MCI patients.

## Introduction

Mild cognitive impairment (MCI), as a multifactorial and complex central neurodegenerative disease, is a phase of predementia with a high conversion rate to Alzheimer’s disease (AD; Petersen, 2016; Anderson, 2019). The clinical feature of MCI is a progressive decline of specific cognitive function (such as memory function, language function, executive function, etc.) that is dependent on the location or cause of brain impairment (Gauthier et al., 2006; Petersen, 2011; Montero-Odasso et al., 2017). In addition, as the current global population is aging, the prevalence of MCI has tripled, increasing the economic burden of MCI control (Vos et al., 2015; Jia et al., 2020). Furthermore, this causes a heavy economic burden on MCI control (Pater, 2011; Lin and Neumann, 2013). Owing to the complex pathogenesis of MCI (Sultana et al., 2009), no disease-modifying therapy is available. Therefore, MCI is regarded as a serious healthcare and economic concern worldwide.

Currently, there is no cure for MCI (Massoud et al., 2007; Wang et al., 2021; Masika et al., 2022). Therefore, MCI is commonly intervened using various pharmacological therapies to improve symptoms and slow the progression. Since mainstay medicines, such as acetylcholinesterase inhibitors and N-methyl-D-aspartate receptor antagonists, produce side effects in patients with MCI (Petersen et al., 2018), many researchers have investigated other methods for controlling the disease (Horr et al., 2015; Rodakowski et al., 2015; Wang Y. Q. et al, 2020). Non-pharmacological therapies are associated with few adverse events and may complement pharmacological therapy or prevent MCI progression; therefore, they are regarded as potential treatments for MCI. Currently, numerous studies have shown that several physical therapies have the potential to benefit patients with MCI (Bachurin et al., 2018; Canu et al., 2018).

Acupuncture, a physical therapy used in China, has been widely applied to ameliorate cognitive, memory, and other types of functional decline for at least the past 2,000–3,000 years (Gao et al., 2019; Zhou et al., 2020; Bao et al., 2021; Su et al., 2021; WuLi et al., 2021). It involves using needle insertion into specific acupoints (skin and underlying tissues) to enhance the cognitive ability of patients. Many systematic reviews (SRs; Cao et al., 2013; Deng et al., 2016; Lai et al., 2020; He et al., 2021; Yin et al., 2022) have demonstrated that acupuncture results in cognitive enhancement in MCI, improved subjective cognitive decline, and diminished post-stroke cognitive impairment without obvious side effects. The positive effects of acupuncture on MCI have encouraged researchers to further explore the therapeutic effects of acupuncture. Nonetheless, the mechanism by which acupuncture promotes cognitive function remains largely unknown.

The central nervous system plays a vital role in corresponding to the cognitive function. Numerous studies (Bajo et al., 2010; Han et al., 2012; Tomasi and Volkow, 2012; Hoffstaedter et al., 2015) have illustrated that the decline of cognitive function is associated with changes in brain areas and networks. However, the decline process in the MCI period maybe reversible because of the human brain’s plasticity and adaptivity (Cohen et al., 2009; Hötting and Röder, 2013). Physical stimulation is an approach to enhancing brain plasticity (Erickson et al., 2013). Considering the crucial role of brain plasticity in MCI, experts, scholars, and researchers have attempted attempt to understand the neural mechanism underlying acupuncture-related improvement in cognitive performance.

With technical advances, numerous noninvasive neuroimaging methods (such as functional magnetic resonance imaging (fMRI), diffusion tensor imaging (DTI), functional near-infrared spectroscopy (fNIRS), magnetic resonance spectroscopy (MRS), etc.) have been applied to identify neural features of acupuncture-induced alternations in patients with MCI (Wang et al., 2012; Liu et al., 2014; Tan et al., 2017; Shan et al., 2018). However, no SR has summarized the neuroimaging evidence regarding using acupuncture for MCI. Therefore, the present study aimed to explore three issues: (1) the main characteristics of current neuroimaging studies; (2) the acupuncture-induced alternations in the brain; and (3) the direction of future study. Thus, we summarized main characteristics, core brain areas, and potential networks involved in the response to acupuncture to better understand the neurological mechanism by which acupuncture improves MCI and provide suggestions and references for future research.

## Materials and methods

This SR was registered on the PROSPERO platform (number: CRD42022331525). The study strictly followed the Preferred Reporting Items for Systematic Review and Meta-Analysis (PRISMA) guidelines (Page et al., 2021).

### Inclusion and exclusion criteria

Trials were incorporated in the assessment if they met the following criteria: (1) original, peer-reviewed neuroimaging clinical studies published in Chinese or English; (2) all patients met the diagnostic criteria of MCI; (3) the intervention group received acupuncture, regardless of acupuncture points, acupuncture methods, acupuncturists, and treatment duration; (4) the control group was healthy control, conventional medicine, sham acupuncture, and others; and (5) the neuroimaging tools used were fMRI, DTI, fNIRS, and/or MRS.

Studies were excluded if they met any of the following criteria: (1) reviews, letters, comments, protocols, and experimental studies; (2) duplicated/retracted articles; and (3) if they reported insufficient/unavailable data.

### Search strategies

The following electronic databases were searched independently by two reviewers: PubMed, Embase, Cochrane Library, Web-of-Science Core Collection, Chinese Biomedical Literature Database, China National Knowledge Infrastructure, VIP Database, WF Database, Gray Literature Database, and other resources (ClinicalTrials.gov, Chinese Clinical Trial Register (ChiCTR), World Health Organization International Clinical Trials Registry Platform (WHO ICTRP)) from the date of database inception to 1 June 2022. The following phrases were used for searches of the literature: (1) clinical condition: mild cognitive impairment, cognitive dysfunction, and MCI; (2) acupuncture terms: acupuncture, electronic acupuncture, acupuncture moxibustion, warm needling, scalp needle, meridian, and acupoint; and (3) study type: neuroimaging trial. Search terms were combined using “and” and “or.” The electronic database search strategies used are presented in Appendix 1.

### Study selection and data extraction

Two investigators (MX and ZC) independently screened identified studies. Intra-class correlation coefficient score (score = 0.95) was applied to evaluate between-investigator consistency. MX and ZC first read the titles and keywords of selected studies to identify duplicate articles. Thereafter, the investigators assessed article titles, abstracts, and keywords, selecting trials based on inclusion criteria. Finally, the investigators screened the full texts of studies to confirm that they met inclusion criteria. Any dispute between the two investigators was handled through an interchange. If no ideal solution was reached, a third referee (LZ or FL) assisted in making a final decision.

Two data extractors (MX and ZC) extracted information using a self-defined standardization extraction form that covered six general topics: (1) identification information (first author’s name and year of publication); (2) basic information (study design, sample size, diagnostic criteria, age, and gender); (3) acupuncture details based on the Revised Standards for Reporting Interventions in Clinical Trials of Acupuncture (STRICTA) MacPherson et al., 2002; (4) details regarding controls used; (5) clinical outcomes; and (6) neuroimaging information. The procedure is displayed using a PRISMA flow diagram.

### Quality assessment

Cochrane’s tools were used to assess the methodological quality. For randomized controlled trials (RCTs), the Risk of Bias 2.0 tool (RoB 2; Sterne et al., 2019) was applied. The risk of bias (RoB) of each study was assessed and classified as follows: high, low, or some concerns. For non-randomized controlled trials (non-RCTs), the Risk Of Bias In Non-randomized Studies of Interventions (ROBINS-I) tool (Sterne et al., 2016) was applied. The overall RoB was classified as follows: critical, serious, moderate, low, or no information. A third party was consulted to resolve any disagreement between investigators.

### Statistical analysis for acupuncture-induced brain alterations

Owing to the various neuroimaging methodology tools used in the included trials, a descriptive statistical analysis was considered appropriate. In addition, a narrative analysis was carried out to summarize the acupuncture-induced structural or functional brain alterations, regardless of acupuncture’s instant/sustained effect.

## Results

### Search and selection of studies

**Figure 1:**
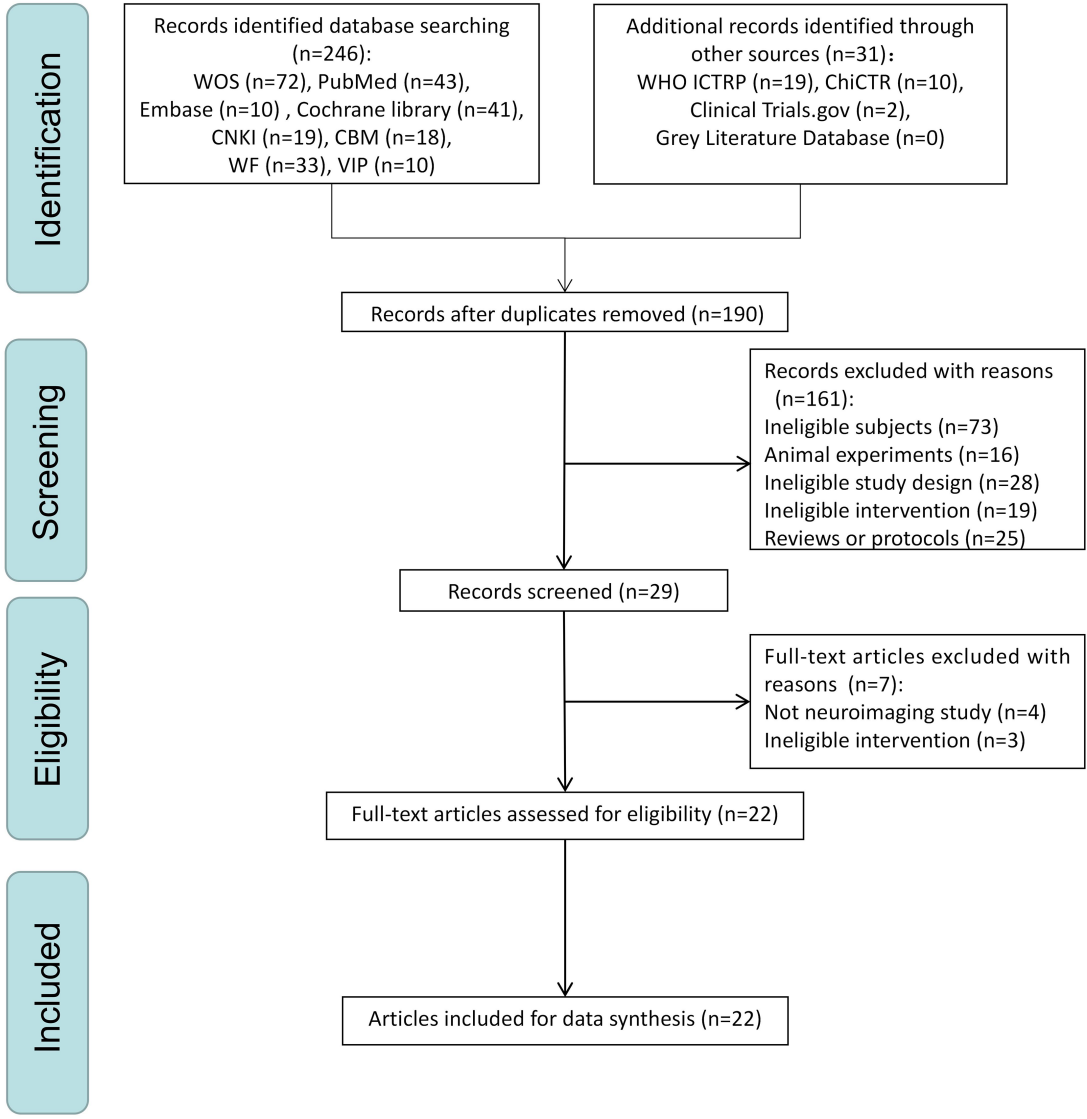
The PRISMA flow chart of selection process.

A PRISMA flow plot describing the methodology used to search and screen trials is shown in Figure 1. A total of 277 studies were included after a comprehensive search was implemented. After deduplication, 190 studies remained. After the initial screening phase, only 29 trials remained. After the second screening phase (screening full-text articles), seven articles were excluded (four that did not include neuroimaging data and three with treatments considered ineligible) according to the inclusion criteria, leaving 22 trials (Liu, 2009, 2010; Jin, 2010; Cui, 2011; Jiang, 2011; Feng et al., 2012; Jiang et al., 2012; Wang et al., 2012; Chen et al., 2013, 2014; Xu, 2013; Xu et al., 2013; Liu et al., 2014; Jia et al., 2015; Tan et al., 2017; Xu and Peng, 2017; Shan et al., 2018; Ghafoor et al., 2019; Li et al., 2020; Wang F. et al., 2020; Cao et al., 2021; Khan et al., 2022) for the final analysis. The reasons for excluding the selected full-text trials are presented in Appendix 2.

### Study characteristics

The key characteristics of the 22 neuroimaging trials included in this review are presented in Table 1. The publication dates of the included trials ranged from 2009 to 2022. In total, 20 studies (90.9%) were conducted in China and 2 (9.1%) were conducted in Korea. Eleven trials (50%) were published in English, whereas the others were published in Chinese.

**Table 1:** Main characteristics of included neuroimaging studies.

Study	Country	Study design	N (A/B/C)	Diagnostic criteria	Age (Year)	Gender (M/F)	Treatment courses	(A)	(B)	(C)	Imaging modality	Scan T	Clinical outcomes	Analytical approaches
								Intervention group	Control group I	Control group II				
Khan et al. (2022)	Korea	Non-RCT	22 (11/11)	/	A:61.58 ± 6.55	A: 10/17	12 weeks	Acupuncture	HC	/	fNIRS	/	MoCA, working memory adaption rate	Brain activation
					B:55.92 ± 7.65	B: 4/16								
Cao et al. (2021)	China	Non-RCT	16	①	68.72 ± 4.03	/	16 min	Acupuncture	/	/	ts-fMRI	3.0 T	/	Brain activation
Wang F. et al., 2020	China	Non-RCT	36	⑥	64.96 ± 3.22	16/20	28 days	Acupuncture	/	/	rs-fMRI	3.0 T	MMSE, MoCA	Amplitude of low-frequency fluctuation
Li et al. (2020)	China	RCT	78 (12/13/50)	③	A:67.5 ± 9.7	A: 8/4	6 months	Acupuncture + B	CM (12 mg/day dose of Rivastigmine)	HC	rs-fMRI	3.0 T	MoCA, MMSE, AVLT, CDR	Functional connectivity, correlation with MoCA
					B:65.0 ± 10.1	B: 7/6								
					C:65.8 ± 7.2	C: 24/26								
Ghafoor et al. (2019)	Korea	Non-RCT	24 (12/12)	/	A:61.58 ± 6.55	/	12 weeks	Acupuncture	HC	/	fNIRS	/	MoCA	Functional connectivity, and correlation with MoCA
					B:55.92 ± 7.65									
Shan et al. (2018)	China	RCT	14 (8/6)	②	A:66.38 ± 10.97	A: 3/5	3 min	Acupuncture	Sham acupuncture	/	ts-fMRI	3.0 T	/	Brain activation
					B: 67.83 ± 6.01	B: 3/3								
Xu and Peng (2017)	China	RCT	60 (30/30)	⑤	A:62.12 ± 8.01	A: 16/14	8 weeks	Acupuncture	CM (60 mg/day dose of Nimodipine)	/	rs-fMRI, DTI	3.0 T	MMSE, MoCA, CDT	Regional homogeneity, fractional anisotropy
					B:61.20 ± 7.63	B: 16/14								
Tan et al. (2017)	China	RCT	32 (16/16)	②	A:65.88 ± 4.66	A: 10/6	4 weeks	Acupuncture	Sham acupuncture	/	rs-fMRI	3.0 T	MMSE, MoCA, DST, ADAS-Cog	Functional connectivity
					B:64.56 ± 5.25	B: 6/10								
Jia et al. (2015)	China	Non-RCT	23 (8/15)	/	A:74.1 ± 7.8	A: 2/6	During fMRI scanning	Acupuncture	HC	/	rs-fMRI	1.5 T	MMSE, ADAS-Cog	Amplitude of low-frequency fluctuation
					B:73.7 ± 7.3	B: 8/7								
Liu et al. (2014)	China	Non-RCT	24 (12/12)	/	A:59.3 ± 3.3	A: 1/11	3 min	Acupuncture	HC	/	rs-fMRI	3.0 T	/	Regional homogeneity
					B:60.6 ± 5.8	B: 4/8								
Chen et al. (2014)	China	Non-RCT	24 (12/12)	②	A:59.3 ± 3.3	A: 1/11	3 min	Acupuncture	HC	/	ts-fMRI	3.0 T		Brain activation
					B:60.6 ± 5.8	B: 4/8								
Xu, 2013	China	RCT	36 (12/12/12)	②④	A:59.3 ± 3.3	A: 8/4	3 min	Acupuncture	Sham acupuncture	HC	ts-fMRI	3.0 T	MoCA, CDR	Brain activation
					B:62.8 ± 5.9	B: 7/6								
					C:60.6 ± 5.8	C: 24/26								
Xu et al., 2013	China	Non-RCT	6	/	55–70	4/2	9 min	Acupuncture	/	/	rs-fMRI	3.0 T	/	Functional connectivity
Chen et al. (2013)	China	Non-RCT	24 (12/12)	/	A:59.3 ± 3.3	A: 1/11	3 min	Acupuncture	HC	/	rs-fMRI	3.0 T	/	Functional connectivity
					B:60.6 ± 5.8	B: 4/8								
Jiang et al. (2012)	China	RCT	24 (12/12)	②④	A:63.83 ± 4.90	A: 6/6	3 min	Acupuncture	Sham acupuncture	/	ts-fMRI	3.0 T	/	Brain activation
					B:67.08 ± 5.26	B: 6/6								
Wang et al. (2012)	China	Non-RCT	22 (8/14)	②	A:66.37 ± 10.96	A: 3/5	3 min	Acupuncture	HC	/	ts-fMRI	3.0 T	MMSE, AVLT, CDR	Brain activation
					B:66.07 ± 5.78	B: 6/8								
Feng et al. (2012)	China	Non-RCT	24 (12/12)	/	A:59.3 ± 3.3	A: 1/11	3 min	Acupuncture	HC	/	rs-fMRI	3.0 T	/	Functional connectivity
					B:60.6 ± 5.8	B: 4/8								
Jiang (2011)	China	Non-RCT	24 (12/12)	②	A:66.83 ± 4.90	A: 6/6	3 min	Acupuncture	HC	/	ts-fMRI	3.0 T	/	Brain activation
					B:62 ± 4.46	B: 5/7								
Cui (2011)	China	RCT	36 (12/12/12)	②④	A:59.3 ± 3.3	A: 3/9	3 min	Acupuncture	Sham acupuncture	HC	ts-fMRI	3.0 T	MMSE, CDR	Brain activation
					B:62.8 ± 5.9	B: 4/8								
					C:60.6 ± 5.8	C: 4/8								
Liu (2010)	China	RCT	36 (17/19)	①	A:66.00 ± 6.84	A: 7/10	30 days	Acupuncture	CM (2.5 mg/day dose of Donepezil)	/	MRS	1.5 T	MMSE, MoCA	Metabolic Ration
					B:69.32 ± 6.86	B: 9/10								
Jin (2010)	China	RCT	30 (14/16)	②	A:72.54 ± 7.07	A: 8/6	45 days	Acupuncture	CM (90 mg/day dose of Nimodipine)	/	MRS	1.5 T	MoCA, CMS	Metabolic Ration
					B:73.67 ± 3.27	B: 9/7								
Liu (2009)	China	RCT	32 (15/17)	①	A:73 ± 7.67	A: 9/6	30 days	Acupuncture	CM (2.5 mg/day dose of Donepezil)	/	MRS	1.5 T	MMSE, CMS	Metabolic Ration
					B:77.22 ± 5.65	B: 9/8								

①: Diagnostic and Statistical Manual of Mental Disorders; ②: Petersen RC criteria; ③: National Institute on Aging and Alzheimer’s Association criteria; ④: Expert consensus on prevention and treatment of Cognitive Impairment in China; ⑤: National Institute of Neurological and Communicative Disorders and Stroke and the Alzheimer’s Disease and Related Disorders Association criteria; ⑥: Guidelines for the diagnosis and treatment of dementia and cognitive impairment in China; RCT, randomized controlled trial; HC, healthy control; CM, conventional medicine; fNIRS, functional near-infrared spectroscopy; ts-fMRI, task-state functional magnetic resonance imaging; rs-fMRI, task-state functional magnetic resonance imaging; DTI, diffusion tensor imaging; MRS, magnetic resonance spectroscopy; MMSE, the Mini-Mental State Examination; MoCA, Montreal Cognitive Assessment Scale; CDR, Clinical Dementia Rating; AVLT, Auditory Verbal Learning Test; ADAS-cog, Alzheimer’s disease assessment scale-cognitive subscale; CMS, Clinical Memory Scale; CDT, Clock Drawing Test; DST, Double Support Time.

### Study design

Regarding the study design, 10 RCTs and 12 non-RCTs were assessed. Sample sizes of the studies ranged from 6 to 78 individuals. Sixteen studies were designed to investigate whether acupuncture induces cerebral responses. In addition, six trials were designed to investigate whether acupuncture affects neural networks in the brain.

### Participants

A total of 511 patients with MCI and 136 healthy controls were included in this assessment. In total, nine neuroimaging trials used Petersen’s criteria, six used other criteria, and seven did not mention the criteria used. Ten neuroimaging trials compared patients with MCI and healthy subjects, whereas others enrolled patients with MCI exclusively. All studies included participants with MCI aged 55–74 years. Twenty studies indicated the sex of patients with MCI (290 male and 380 female). Two studies did not report the sex of the patients with MCI. The sample sizes of the 10 articles that enrolled patients with MCI exclusively ranged from 6 to 36 per group. Among 12 trials that compared MCI patients and healthy volunteers, the main matching sample size ratio of MCI/healthy control was 1/1.

### Acupuncture details

Based on the Standards for Reporting Interventions in Clinical Trials of Acupuncture (STRICTA) guidelines, acupuncture details are summarized and presented in Table 2. The rationale (acupuncture type and the reason for treatment provided) for selecting a particular type of acupuncture was mentioned in all neuroimaging trials. The number of needle insertions varied from 1 to 17 per session for each subject. KI 3 (Taixi), LR 3 (Taichong), and LI 4 (Hegu) were the acupoints most often used. Acupuncture insertion depth was 5–30 mm. Only eight neuroimaging trials described the deqi sensation. Manual acupuncture was applied in 18 articles, and electronic acupuncture was used in four studies. The diameter and length of the needles used in the included studies were 0.35 and 25 mm, respectively. The number of treatment sessions ranged from 1 to 48. Most frequently, one 3-min session was performed. Only six neuroimaging trials provided information about the acupuncturists. In total, 18 trials provided an elaborate depiction.

**Table 2:** Details of acupuncture methods according to Standards for Reporting Interventions in Clinical Trials of Acupuncture (STRICTA).

Study	Acupuncture rationale			Details of needling							Treatment regimen		Other components		Practitioner	Comparator interventions	
	1a	1b	1c	2a	2b	2c	2d	2e	2f	2 g	3a	3b	4a	4b	5	6a	6b
Khan et al. (2022)	TKM	Y	NA	14	GV 20, EX-HN 1, CV 12, HT 7 (bilateral), ST 36 (bilateral), SP 6 (bilateral), KI3 (bilateral)	5–10 mm	No	Manual	10 min	Diameter and length: 0.20 & 30 mm	24	Frequency: twice per week	NA	NR	Y	Y	Y
										Needle brand: Dongbang		Duration: 12 weeks					
Cao et al. (2021)	TCM	Y	NA	1	KI 3 (unilateral, right)	10–17.5 mm	NR	Manual	8 min	NR	1	Frequency: once	NA	NR	Y	NA	NA
												Duration: 8 min					
Wang F. et al., 2020	TCM	Y	NA	17	LR 3 (bilateral), HT 7 (bilateral), SP 3 (bilateral), ST 40 (bilateral), KI 3 (bilateral), BL 58 (bilateral), GV 20, CV 4, GB 13 (bilateral), GB 20 (bilateral)	NR	Deqi	Manual	40 min	NR	24	Frequency: six times per week	NA	NR	NR	NA	NA
												Duration: 28 days					
Li et al. (2020)	TCM	Y	NA	4	LR 3 (bilateral), LI 4 (bilateral)	20 mm	Deqi	Manual	20 min	Diameter and length: 0.30 & 25 mm	48	Frequency: three times per week	NA	NR	NR	Y	Y
										Needle brand: NR		Duration: 6 months					
Ghafoor et al. (2019)	TKM	Y	NA	14	GV 20, EX-HN 1, CV 12, HT 7 (bilateral), ST 36 (bilateral), SP 6 (bilateral), KI3 (bilateral)	5–10 mm	No	Manual	10 min	Diameter and length: 0.20 & 30 mm	24	Frequency: twice per week	NA	NR	Y	Y	Y
										Needle brand: Dongbang							
												Duration: 12 weeks					
Shan et al. (2018)	TCM	Y	NA	4	LR 3 (bilateral), LI 4 (bilateral)	NR	NR	Manual	3 min	NR	1	Frequency: once	NA	NR	NR	Y	NR
												Duration: 3 min					
Xu and Peng (2017)	TCM	Y	NA	8	GV 20, EX-HN 1, GV 24, GB 20 (bilateral)	12.5–30 mm	Deqi	Electronic	NR	Diameter and length: 0.30 & 40 mm	24	Frequency: three times per week	NA	NR	NR	Y	Y
										Needle brand: Zhongyan							
										Electroacupuncture apparatus: 6805C		Duration: 20 min					
Tan et al. (2017)	TCM	Y	NA	13	EX-HN1, GV 29, PC 6 (bilateral), KI 3 (bilateral), ST 40 (bilateral), and LR 3 (bilateral)	15 mm	NR	Manual	NR	Diameter and length: 0.35 & 25 mm	20	Frequency: five times per week	NA	NR	Y	Y	Y
										Needle brand: Hwato		Duration: 12 weeks					
Jia et al. (2015)	TCM	Y	NA	2	KI3 (bilateral)	20 mm	NR	Manual	NR	Diameter and length: 0.25 & 40 mm	1	Frequency: once	NA	NR	NR	Y	Y
												Duration: NR					
										Needle brand: Hwato							
Liu et al. (2014)	TCM	Y	NA	1	KI 3 (unilateral, right)	10–22 mm	NR	Manual	3 min	Diameter and length: 0.20 & 40 mm	1	Frequency: once	NA	NR	Y	Y	Y
												Duration: 3 min					
										Needle brand: NR							
Chen et al. (2014)	TCM	Y	NA	1	KI 3 (unilateral, right)	12 mm	NR	Manual	3 min	Diameter and length: 0.35 & 25 mm	1	Frequency: once	NA	NR	Y	Y	Y
										Needle brand: Hwato		Duration: 3 min					
Xu, 2013	TCM	Y	NA	1	KI 3 (unilateral, right)	10 mm	NR	Manual	3 min	Diameter and length: 0.35 & 25 mm	1	Frequency: once	NA	NR	NR	Y	Y
												Duration: 3 min					
										Needle brand: Hwato							
Xu et al., 2013	TCM	NR	NA	1	GV 26	7.5–12.5 mm	NR	Manual	9 min	Diameter and length: 0.35 & 25 mm	1	Frequency: once	NA	NR	NR	NA	NA
										Needle brand: NR		Duration: 9 min					
Chen et al. (2013)	TCM	Y	NA	1	KI 3 (unilateral, right)	10–20 mm	Deqi	Manual	3 min	Diameter and length: 0.35 & 25 mm	1	Frequency: once	NA	NR	NR	Y	Y
												Duration: 3 min					
										Needle brand: Hwato							
Jiang et al. (2012)	TCM	Y	NA	1	KI 3 (unilateral, right)	15 mm	NR	Manual	3 min	Diameter and length: 0.35 & 25 mm	1	Frequency: once	NA	NR	NR	Y	Y
												Duration: 3 min					
										Needle brand: NR							
Wang et al. (2012)	TCM	Y	NA	4	LR 3 (bilateral), LI 4 (bilateral)	NR	NR	Manual	3 min	Diameter and length: 0.30 & 25 mm	1	Frequency: once	NA	NR	NR	Y	Y
												Duration: 3 min					
										Needle brand: NR							
Feng et al. (2012)	TCM	Y	NA	1	KI 3 (unilateral, right)	10–20 mm	Deqi	Manual	3 min	Diameter and length: 0.2 & 40 mm	1	Frequency: once	NA	NR	NR	Y	Y
												Duration: 3 min					
										Needle brand: NR							
Jiang (2011)	TCM	Y	NA	1	KI 3 (unilateral, right)	15 mm	NR	Manual	3 min	Diameter and length: 0.30 & 25 mm	1	Frequency: once	NA	NR	NR	Y	Y
												Duration: 3 min					
										Needle brand: NR							
Cui (2011)	TCM	Y	NA	1	KI 3 (unilateral, right)	10 mm	NR	Manual	3 min	NR	1	Frequency: once	NA	NR	NR	Y	Y
												Duration: 3 min					
Liu (2010)	TCM	Y	NA	9	GV 20, GB 20 (bilateral), BL 23(bilateral), GB 39 (bilateral), KI3 (bilateral)	13–25 mm	Deqi	Electronic	NR	Diameter and length: 0.35 & 25 mm	30	Frequency: each day	NA	NR	NR	Y	Y
										Needle brand: Shenlong							
												Duration: 30 days					
										Electroacupuncture apparatus: G6805-II							
Jin (2010)	TCM	Y	NA	10	EX-HN 1, GB 20 (bilateral), BL 23(bilateral), KI3 (bilateral)	13–25 mm	Deqi	Electronic	20 min	Diameter and length: 0.30 & 40 mm	45	Frequency: each day	NA	NR	NR	Y	Y
										Needle brand: Hwato							
												Duration: 45 days					
										Electroacupuncture apparatus: G6805-II							
Liu (2009)	TCM	Y	NA	12	GV 20, GB 20 (bilateral), BL 23(bilateral), GB 39 (bilateral), KI3 (bilateral), HT 7 (bilateral)	7.5–25 mm	Deqi	Electronic	20 min	Diameter and length: 0.30 & 25 mm	30	Frequency: each day	NA	NR	NR	Y	Y
												Duration: 30 days					
										Needle brand: Shenlong							
										Electroacupuncture apparatus: G6805-II							

1a: Style of acupuncture; 1b: Reasoning for treatment provided; 1c: Extent to which treatment was varied; 2a: Number of needle insertions per subject per session; 2b: Names of points used; 2c: Depth of insertion; 2d: Response sought; 2e: Needle stimulation; 2f: Needle retention time; 2 g: Needle type; 3a: Number of treatment sessions; 3b: Frequency and duration of treatment sessions; 4a: Details of other interventions administered to the acupuncture group; 4b: Setting and context of treatment; 5: Description of participating acupuncturists; 6a: Rationale for the control or comparator; 6b: Precise description of the control or comparator; TCM, traditional Chinese medicine; TKM, traditional Korean medicine; NA, not application; NR, not recorded; Y, Yes; &, and.

### Comparisons performed

Among the 22 articles considered, the following five comparison models were used: acupuncture vs. healthy volunteers (*n* = 11 trials), self-control model (pre- vs. post-treatment, *n* = 3 trials), acupuncture plus conventional medicine vs. conventional medicine (*n* = 1 trial), acupuncture vs. conventional medicine (*n* = 5 trials), and acupuncture vs. sham acupuncture (*n* = 5 trials).

### Clinical outcomes

Studies used the following eight cognitive assessment tools to evaluate MCI severity: Mini-Mental State Examination (MMSE; nine studies; 40.9%), Montreal Cognitive Assessment Scale (MoCA; eight studies; 36.36%), Clinical Dementia Rating (CDR; four studies; 18.18%), Auditory Verbal Learning Test (AVLT; two studies; 9.09%), Alzheimer’s disease assessment scale-cognitive subscale (ADAS-cog; two studies; 9.09%), Clinical Memory Scale (CMS; two studies; 9.09%), Clock Drawing Test (CDT; one study; 4.55%), and Double Support Time (DST; one study; 4.55%).

### Imaging conditions and analyses

**Figure 2:**
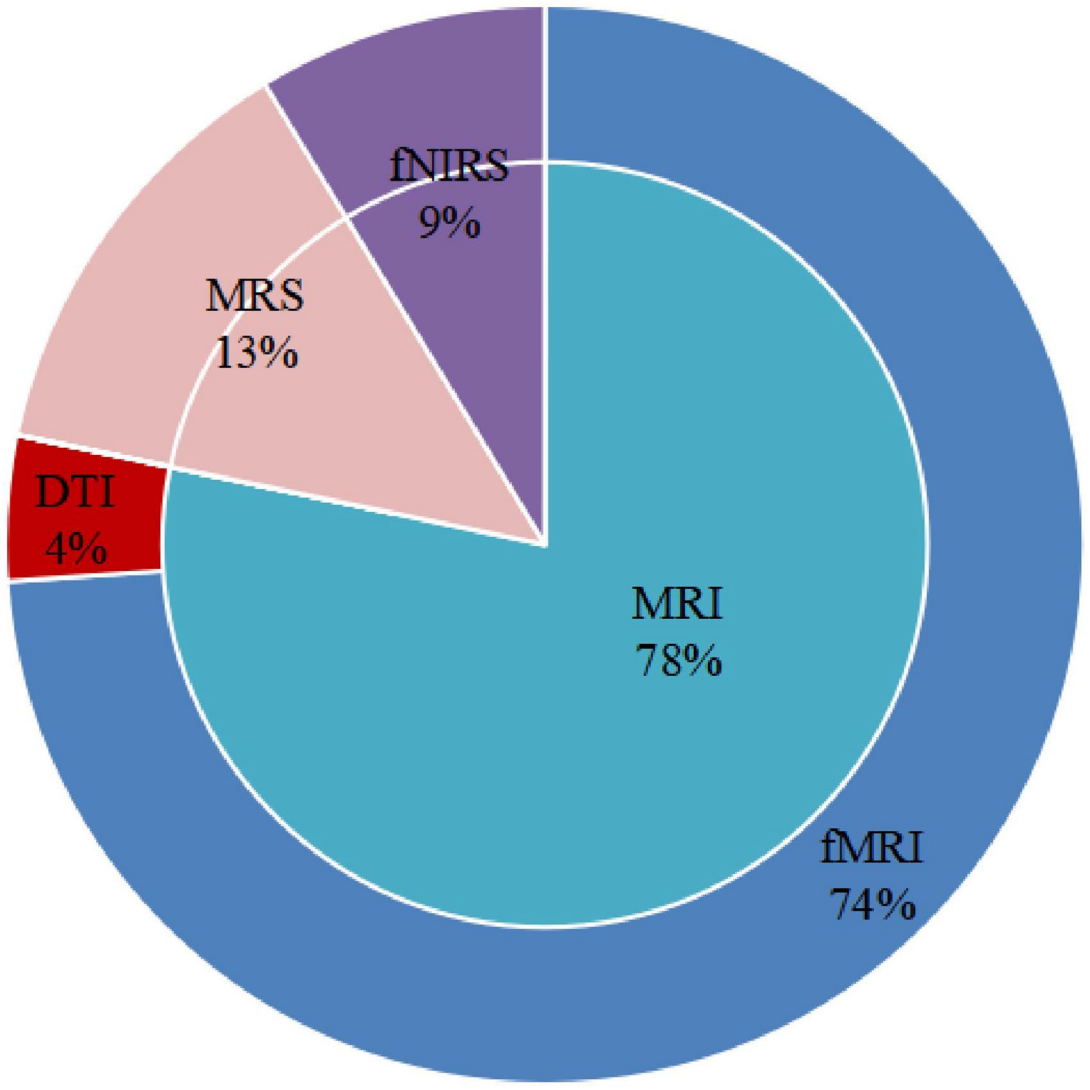
The proportion of scanning techniques.

fMRI, DTI, fNIRS, and MRS were used to explore neuronal activities, functional brain alterations, brain structural alterations, and the metabolic ratio induced by acupuncture in patients with MCI. Only one trial assessed structural changes. The study carried out DTI to explore fractional anisotropy (FA). Three articles evaluated the metabolic ratio using MRS. One study evaluated hemodynamic responses *via* fNIRS, and another investigated functional connectivity (FC). Seventeen studies measured the functional changes induced by acupuncture. Eight studies used task-state functional magnetic resonance imaging (ts-fMRI) to measure cerebral neuron alterations. Eight studies used a single-block design. Acupuncture involved a persistent stimulation for 3 or 16 min per block. Nine studies employed resting-state functional magnetic resonance imaging (rs-fMRI) to investigate FC (five studies), regional homogeneity (ReHo; two studies), or amplitude of low-frequency fluctuation (ALFF; two studies). Figure 2 displays the proportions of imaging conditions and analytical methods used.

### Quality assessment

The results of methodological quality assessments are depicted in Appendices 3, 4.

Among the 10 RCTs assessed, a moderate RoB was found in all studies using the RoB 2 tool. With regard to randomization, there was some concern with all articles because they all had ill-defined random sequence generation methods. Five articles had low bias regarding deviation from the intended intervention, whereas there was some concern with five studies due to short descriptions. Only one study had notable missing outcome data. The others were determined to have low bias. Notably, all articles were considered at a low RoB regarding outcome measurements. There were concerns with all RCTs regarding the selection of reported results because of a lack of protocol and registration information.

Of the 12 non-RCTs considered, a moderate RoB for five studies and a low RoB for seven studies were shown using the ROBINS-I tool. Regarding confounders, seven articles had a low bias, whereas five articles had a moderate RoB due to missing or mixed information. Notably, all articles demonstrated low bias with regard to participant selection, the classification of interventions deviating from those intended, missing data, outcome measurement, and the selection of reported results.

### Neuroimaging results

#### Potential neural mechanisms underlying acupuncture

According to the studies considered, acupuncture-induced brain alterations in MCI patients occurred principally in the cingulate cortex (eight studies), hippocampus (six studies), or prefrontal cortex (six studies). The brain regions that were reported in the included studies were key regions of the default mode network (DMN; Raichle, 2015; Smallwood et al., 2021), central executive network (CEN; Chen et al., 2019; Fang et al., 2021; Daigle et al., 2022), and salience network (SN; Chand et al., 2017; Porto et al., 2018; Xue et al., 2021). As acupuncture effects are classified as either constant or instant, findings associated with each were considered separately.

#### Cerebral constant response to acupuncture

As displayed in Appendix 5, the most commonly reported constant acupuncture-related brain alterations in patients with MCI were located in the hippocampus (four studies), prefrontal cortex (four studies), parahippocampal gyrus (two studies), and cingulate cortex (two studies).

Two studies employing fNIRS (Ghafoor et al., 2019; Khan et al., 2022) revealed that that constant acupuncture can improve the hemodynamic response (Khan et al., 2022) and FC in the prefrontal cortex in patients with MCI (Ghafoor et al., 2019). In addition, three trials (Liu, 2009, 2010; Jin, 2010) employing MRS revealed changes in N-acetyl aspartate/creatine, choline/creatine, and myo-inositol/creatine ratios in the temporal gyrus and hippocampus of patients with MCI due to regular acupuncture.

Only one study (Xu and Peng, 2017) reported regional structural changes due to acupuncture treatment. Here, after 24 sessions of acupuncture over 8 weeks, white matter FA was increased in the splenium of the corpus callosum, cingulate gyrus, inferior frontal-occipital, and superior longitudinal fasciculus.

Based on fMRI trials, two neuroimaging studies reported increased ReHo or ALFF after constant acupuncture in brain areas concerned with the processing of cognitive function including memory regions (e.g., the parahippocampal gyrus, temporal lobe, precuneus), visual–spatial regions (e.g., the occipital lobe, lingual gyrus), and affective-emotional processing cognitive function areas (e.g., the insula, cingulate cortex, thalamus). Conversely, one study (Xu and Peng, 2017) revealed that ReHo decreased after acupuncture therapy in brain areas involved in processing cognitive function, including memory regions (e.g., the inferior frontal gyrus, temporal lobe) and executive/language function regions (e.g., the posterior cerebellar lobe, inferior temporal gyrus).

Through the FC matrix, one study (Tan et al., 2017) revealed increased connectivity between cognition-related brain areas such as the hippocampus, insula, dorsolateral prefrontal cortex, thalamus, inferior parietal lobule, and anterior cingulate cortex due to regular acupuncture. In addition, using region of interest-wise (ROI-wise) FC analysis, Li et al. (2020) found that right hippocampal FC with the right inferior temporal gyrus/middle temporal gyrus was significantly enhanced after acupuncture treatment. Furthermore, there was a significant correlation between the FC strength of the right hippocampus–inferior temporal gyrus and MoCA score change.

**Figure 3:**
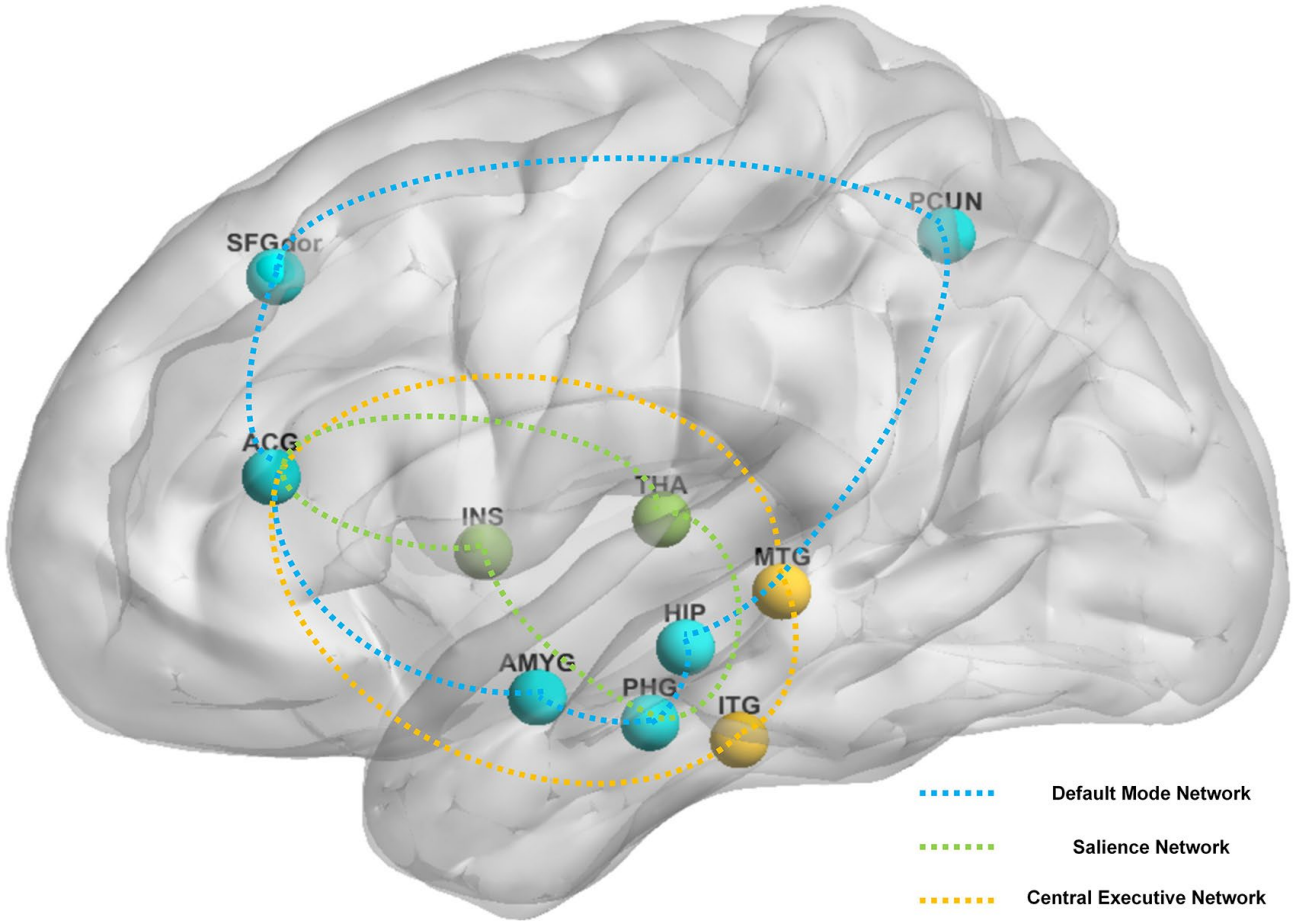
The main reported alternation of brain regions and networks by sustained acupuncture across the studies reviewed. Brain regions: ACG, anterior cingulate and paracingulate gyri; AMYG, amygdala; HIP, hippocampus; INS, insula; ITG, inferior temporal gyrus; MTG, middle temporal gyrus; PCUN, precuneus; PHG, parahippocampal gyrus; SFGdor, superior frontal gyrus, dorsolateral; THA, thalamus. Brain networks: blue network, default mode network; green network, salience network; yellow network, central executive network.

Brain regions reported in the included studies can be roughly classified as regions for three of the following pathways: DMN (e.g., dorsolateral superior frontal gyrus, precuneus, middle temporal gyrus, hippocampus, amygdala, anterior cingulate and paracingulate gyri, parahippocampal gyrus), CEN (e.g., anterior cingulate and paracingulate gyri, inferior temporal gyrus, middle temporal gyrus), and SN (e.g., anterior cingulate and paracingulate gyri, insula, parahippocampal gyrus, thalamus). Figure 3 shows key findings after constant acupuncture.

#### Cerebral instant response to acupuncture

As demonstrated in Appendix 6, fMRI was used in all studies to measure the instant cerebral response to acupuncture in patients with MCI. The most commonly reported brain alterations in MCI subjects undergoing fMRI were in the cingulate cortex (six studies), medial frontal gyrus (five studies), and posterior central gyrus (five studies).

Seven studies explored which brain areas were activated or inactivated after acupuncture. The main brain areas involved included the executive/language function region (e.g., the medial frontal gyrus, middle frontal gyrus), sensory function region (e.g., the posterior central gyrus), affective-emotional processing areas of cognitive function region (e.g., the insula, cingulate cortex), and auditory speech area (e.g., the superior temporal gyrus). In addition, two articles (Liu et al., 2014; Jia et al., 2015) reported increased ReHo or ALFF after instant acupuncture in brain areas involved in the processing of cognitive function, including the memory regions (e.g., the parahippocampal gyrus, precuneus), affective-emotional processing areas of cognitive function (e.g., the cingulate cortex, thalamus), and executive/language function region (e.g., the middle frontal gyrus).

Through FC analysis, Xu et al. (2013) demonstrated increased connectivity between the dorsal lateral prefrontal cortex and frontal and bilateral frontal lobes due to instant acupuncture, and decreased connectivity between the bilateral inferior parietal lobule after instant acupuncture. In addition, using whole-brain FC analysis, Feng et al. (2012) found that connections among the hippocampus, amygdala, parahippocampal gyrus, insula, and cingulate cortex are significantly enhanced after acupuncture. In addition, *via* using multivariate granger causality analysis (mGCA) to assess connectivity it was shown that the dorsolateral prefrontal cortex and hippocampus act as central hubs and significantly influence each other.

**Figure 4:**
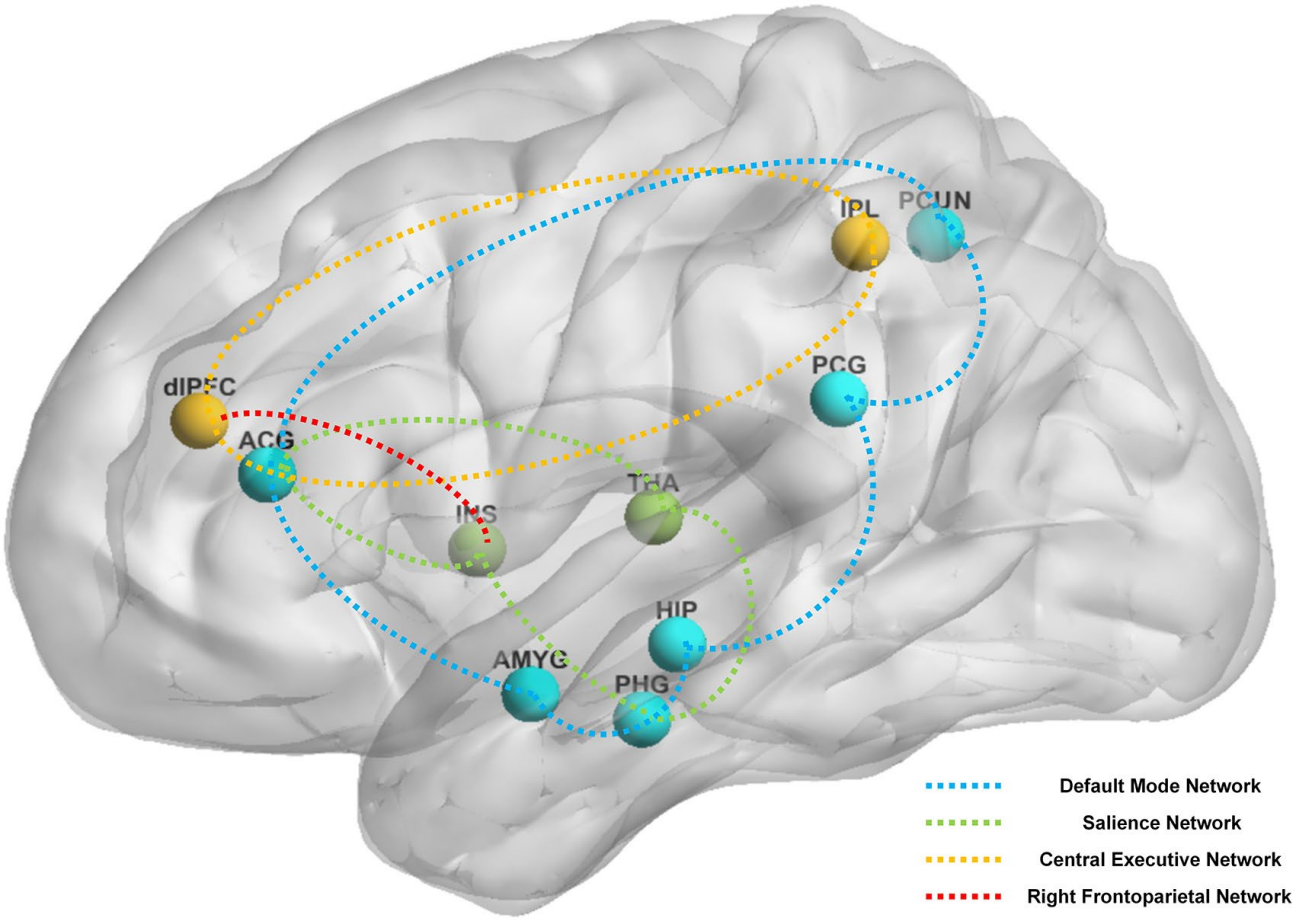
The main reported alternation of brain regions and networks by instant acupuncture across the studies reviewed. Brain regions: ACG, anterior cingulate and paracingulate gyri; AMYG, amygdala; dlPFC, dorsolateral prefrontal cortex; HIP, hippocampus; INS, insula; IPL, Inferior parietal, but supramarginal and angular gyri; PCG, posterior cingulate gyrus; PCUN, precuneus; PHG, parahippocampal gyrus; THA, thalamus. Brain networks: blue network, default mode network; green network, salience network; yellow network, central executive network; red network, right frontoparietal network.

Brain areas reported in the above studies can be roughly classified based on their involvement in the following four pathways: DMN (e.g., posterior cingulate gyrus, anterior cingulate and paracingulate gyri, precuneus, hippocampus, amygdala, parahippocampal gyrus), CEN (e.g., dorsolateral prefrontal cortex, anterior cingulate and paracingulate gyri, inferior parietal lobule), SN (e.g., anterior cingulate and paracingulate gyri, insula, parahippocampal gyrus, thalamus), and right frontoparietal network (e.g., dorsolateral prefrontal cortex, insula). Figure 4 shows the main instant effects of acupuncture that were revealed.

## Discussion

Twenty-two articles that used various neuroimaging tools to investigate the neurocentral mechanism of acupuncture were included in this review. Since 2009, the use of neuroimaging methods to investigate the central nervous regulatory mechanism by which acupuncture can affect MCI has gradually attracted attention. This SR focuses on summarizing the characteristics and findings of neuroimaging trials that investigated the effects of acupuncture on MCI to deepen our understanding of the central mechanism by which this occurs.

Regarding study design, among the 22 neuroimaging studies, only 10 were RCTs, and the others were non-RCTs. Meanwhile, only seven had high methodological quality. Thus, to ensure that acupuncture therapy programs that are applied in neuroimaging trials are effective for MCI treatment, it is recommended that RCTs are used to this. More neuroimaging studies with random designs should be conducted to improve the quality of evidence. Meanwhile, the design of future research should be formulated based on the guideline from the Cochrane Handbook for Systematic Reviews of Interventions (Bian et al., 2011; Cumpston et al., 2019). Additionally, the sample size of each study included in our analysis was less than 80. These small sample sizes may undermine the reliability and replicability of expected effect sizes in neuroscience (Moayedi et al., 2018). Thus, enlarging sample sizes by implementing a standardized program has the potential to improve the statistical power of the findings. Moreover, 16 of the studies were designed to investigate whether acupuncture induces cerebral responses. However, whether the studies performed correlation analyses between cerebral responses and clinical outcomes or not, remains unclear. In addition, six of the trials were designed to investigate whether acupuncture affects neural networks in the brain and only two trials explored the relationship between clinical efficacy and FC strength. Thus, future studies should explore the relationship between clinical efficacy and neurological alteration to better understand the neural mechanisms underlying acupuncture-related improvement in cognitive performance.

Nine of the included studies used Petersen’s criteria. The criteria is commonly used to achieve a clinical diagnosis of MCI. Nevertheless, owing to its distinct phenotype and precise diagnosis, the Jak/Bondi criteria (Bondi et al., 2014) are considered a better diagnostic tool than the Petersen’s criteria. Moreover, cognitive functions could be subdivided as follows: memory, executive, and verbal. However, no study has used neuroimaging to investigate the mechanism of acupuncture on specific cognitive domains in MCI. Therefore, the central mechanism by which acupuncture affects an MCI subtype requires investigation using the Jak/Bondi criteria. In addition, in the studies, there were more women than men with MCI. Based on current studies, sex is an important feature affecting pathological mechanisms and treatments for patients with MCI. However, no study investigated sex-disaggregated neuroimaging on the mechanism of acupuncture in MCI. Therefore, sex-disaggregated neuroimaging studies of acupuncture in patients with MCI are required (Overton et al., 2019).

In terms of acupuncture details, 18 studies used manual acupuncture. However, only six trials mentioned the details of the acupuncturists. Therefore, despite the common use of manual acupuncture, it is worth noting that stimulation is difficult to quantify for various manipulations by different acupuncturists. To ensure repeatability and consistency of findings, researchers should formulate elaborate acupuncture procedures and conduct standardized training for acupuncturists at the start of each trial. Furthermore, based on the traditional Chinese medicine theory, the deqi sensation plays a core role in the effect of acupuncture; however, it was reported in fewer than half of the studies. Moreover, numerous neuroimaging trials have illustrated the cerebral response to deqi sensations. Thus, these items have been recorded in detail. Finally, differences in acupuncture rationale, details of needling, treatment regimen, practitioners, comparator interventions, and other details may have influenced the findings of the included studies. Therefore, there is an urgent need to standardize acupuncture procedures based on STRICTA guideline.

The top three comparison models used to compare groups included acupuncture vs. healthy volunteers, acupuncture vs. conventional medicine, and acupuncture vs. sham acupuncture. The acupuncture model vs. healthy volunteers was used to investigate various cerebral activity differences observed when healthy individuals and those with MCI were compared. Models of acupuncture vs. conventional medicine/sham acupuncture were compared to investigate various cerebral activity differences after acupuncture vs. medicine/placebo. However, these models are far from adequate for exploring the mechanism by which acupuncture affects MCI. For instance, according to the STRICTA criteria, the depth, response sought, acupuncture stimulation, practitioners, and other factors affecting acupuncture efficacy require further research.

The most commonly applied cognitive assessments were the MoCA and MMSE. Both tools have been shown to be accurate for the detection of AD. Compared with the MMSE, the MoCA was more commonly used for the identification of MCI. The MoCA is widely used in Western countries and is recommended for evaluating MCI (Beath et al., 2018). However, a common problem with use of MoCA in developing countries is its applicability for evaluating illiterate and lower-educated older adults. MoCA-basic (MoCA-B) is a revised version of the MoCA scale that is especially appropriate for application in older adult subjects who are illiterate or have little education (Huang et al., 2018). Therefore, MoCA-B is recommended for evaluating MCI in developing countries. The AVLT (Crawford et al., 1989) is the most used assessment approach for evaluating episodic memory. The AVLT tool (Xu et al., 2020) includes an assessment of immediate word recall, short-delayed recall, long-delayed recall, cued recall, and recognition. Episodic memory impairment is the most effective predictor of AD. Thus, the AVLT is recommended for measuring episodic memory function in patients with amnestic MCI induced by AD.

To assess cerebral responses to acupuncture in the treatment of MCI, the following neuroimaging methods were applied in the reviewed studies: fMRI, DTI, fNIRS, and MRS. fMRI was the most frequently applied method to investigate the cerebral responses of acupuncture for MCI (Glover, 2011). fMRI indirectly evaluates brain alterations based on the presence of deoxyhemoglobin in venules, the blood oxygenation level-dependent (BOLD) effect. DTI is an advanced MRI technique and is used to provide qualitative and quantitative white matter microarchitecture information (Meoded and Huisman, 2019). fNIRS is based on optical absorption in the brain and is used to monitor functional brain activity changes (Pinti et al., 2020). MRS is an approach used to evaluate levels of specific neurotransmitters and investigate metabolite alterations in the brain (van Ewijk et al., 2015). According to previous studies (Gerardin et al., 2009), MCI is a multidimensional central nervous system disease that that affects brain structure and function. It is well known that these neuroimaging approaches have their own characteristics, so integrating multiple approaches allows for a more comprehensive assessment of the effects of acupuncture on MCI. Among the 22 trials considered, only one study integrated fMRI and DTI findings when investigating cerebral neuron alterations during acupuncture for MCI. The other studies focused either on structural changes in the brain or functional architecture using a single neuroimaging approach. Thus, the application and promotion of multimodal neuroimaging techniques (such as fMRI with DTI, fNIRS with fMRI, and fMRI with MRS) in future research is urgently needed to more comprehensively investigate mechanistic responses to acupuncture. Additionally, other neuroimaging techniques, such as positron emission tomography, electroencephalography, and magnetoencephalography, are approaches that will expand the current knowledge of MCI (Jackson and Snyder, 2008; López et al., 2014; Chandra et al., 2019). Thus, additional neuroimaging techniques are needed to more extensively investigate the mechanistic responses to acupuncture.

Notably, the common brain areas affected by acupuncture were the cingulate cortex, hippocampus, and prefrontal cortex. The cingulate cortex is important for cognitive networks. Accumulating evidence suggests that improving our understanding of the cingulate cortex may likewise enhance our understanding the brain mechanisms underlying MCI (Cera et al., 2019; Jeong et al., 2021). Meanwhile, numerous studies (Pennanen et al., 2004; Xue et al., 2019; Valdés Hernández et al., 2020) have demonstrated that the hippocampus is a brain region impacted by MCI and is closely related with memory and orientation. In addition, the prefrontal cortex is the region responsible for impaired recollection of working memory in MCI (Serra et al., 2022; You et al., 2022). However, no previous study investigated the effect of acupuncture on MCI in a specific brain area. Based on this, this SR suggests researchers should explore the association between the neurological effect of acupuncture for MCI and the specific regions (such as cingulate cortex, hippocampus, and prefrontal cortex) of the brain.

Moreover, neuroimaging studies assessing the neurological effects of acupuncture on MCI has illustrated that acupuncture may regulate brain networks. Important pathways associated with MCI improvement due to acupuncture are summarized as follows. Pivotal areas of the DMN (cingulate cortex, prefrontal cortex, hippocampus, precuneus, and insula), SN (anterior cingulate cortex, insula, parahippocampal gyrus, thalamus), and CEN (anterior cingulate cortex, inferior temporal gyrus, and middle temporal gyrus) mediate cerebral changes in response to acupuncture-based MCI therapy. The DMN plays an important role in cognitive functioning (memory, attention, executive function, and language). The CEN affects cognitive control, episodic memory, etc. The SN is part of an integral mediation hub that affects dynamic interactions between the DMN and CEN networks. Numerous studies have demonstrated that altered triple-network models (DMN, SN, and CEN) are the prominent hallmarks of both AD and MCI (Meng et al., 2022). Further, multiple neuroimaging trials have suggested that regulating the activities of triple-network models is a key mechanism by which acupuncture therapy affects brain functioning (Bai et al., 2009; Deng et al., 2016; Wang et al., 2019). In this SR, most triple-network areas were shown to be involved in the response to acupuncture in patients with MCI, implying that acupuncture may modulate MCI distribution networks. However, up to now, none of the included neuroimaging studies have explored the effect of acupuncture on MCI through the triple-network models. Thus, future, related neuroimaging studies are proposed to investigate the certain effects of acupuncture on MCI in regulating these brain networks.

It has been acknowledged that the effect of acupuncture could be divided into constant effect and the instant effects. In the reviewed studies, researchers not only focused on the instant effect but also the constant effect of acupuncture. We found that the cingulate cortex was the main brain area affected by the acupuncture response, regardless of whether the effects were instant or constant. Thus, the cingulate cortex maybe a crucial structure in the functional response to acupuncture. Additionally, instant but not constant effects of acupuncture in MCI were observed in areas of the right frontoparietal network. Previous studies (Pupíková et al., 2021) have shown that the right frontoparietal network plays a vital role in visual working memory performance. The network may mediate instant effects of acupuncture to improve memory. However, these neuroimaging findings need to be validated.

To the best of our knowledge, current SRs investigating the use of acupuncture for treating MCI have focused primarily on the efficacy and safety of the therapy. However, no SRs have explored the mechanism by which acupuncture affects MCI. As the number of neuroimaging studies on acupuncture for MCI have increased, multiple imaging modalities and various analytical approaches have provided direct evidence of the central neural mechanism for acupuncture therapy in MCI (Liu et al., 2021; Wen et al., 2021). Thus, this SR aims to provide specific insights regarding the neurocentral mechanism by which MCI is alle*via*ted *via* acupuncture by summarizing the current clinical neuroimaging findings.

This study has several limitations. First, various imaging modalities and analytical approaches have been applied, making a complete quantitative meta-analysis difficult. Second, due to the variability regarding acupuncture details (such as acupoints, frequency, treatment session, and needle type), no included articles completely adhered to the STRICTA statement. This has the potential to increase heterogeneity and the risk of bias. Further, the limited number of high-quality studies published may have limited our findings. Considering the instability arising from small sample sizes that have been discussed, the findings should be interpreted with caution.

## Conclusion

Our systematic review summarizes neuroimaging data used to investigate the cerebral response to acupuncture in patients with MCI. The brain areas covered in acupuncture for MCI are mainly located in the DMN, CEN, and SN, especially in the cingulate cortex, hippocampus, and prefrontal cortex. However, the included studies are in the preliminary exploration stage. Thus, multicenter, large sample, and strictly designed RCTs employing multimodal neuroimaging approaches are needed to confirm current neuroimaging findings.

## Data availability statement

The original contributions presented in the study are included in the article/Supplementary material, further inquiries can be directed to the corresponding authors.

## Author contributions

ZY, ZW, and FL conceived this study. ZY and JZ developed and achieved the review, under the supervision of LZ and wrote the first draft of the current review with XL, HY, MS, LZ, ZW. ZY and XZ provided the analysis plan and performed analysis. MX and ZC performed study search, screening, and extraction of data, whereas YL reviewed the work. FL provided input to the final draft. All authors contributed to the article and approved the submitted version.

## Funding

This work was financially supported by the State Administration of Traditional Chinese Medicine, National Natural Science Foundation of China (nos. 81590951, 82004486, 81722050, and 81973961).

## Conflict of interest

The authors declare that the research was conducted in the absence of any commercial or financial relationships that could be construed as a potential conflict of interest.

## Publisher’s note

All claims expressed in this article are solely those of the authors and do not necessarily represent those of their affiliated organizations, or those of the publisher, the editors and the reviewers. Any product that may be evaluated in this article, or claim that may be made by its manufacturer, is not guaranteed or endorsed by the publisher.
