# Supplementary material for: Acupuncture on mild cognitive impairment: A systematic review of neuroimaging studies
Source: Front Aging Neurosci. 2023 Feb 15;15:1007436. doi: 10.3389/fnagi.2023.1007436 (PMC9975578; doi:10.3389/fnagi.2023.1007436)
Supplement: Supplementary file 1 [file Table_1.DOCX]

**Appendix 1. Search strategies of each database.**

**PubMed**

#1 "Cognitive Dysfunction"[Mesh]

#2 "Cognitive Dysfunction" [Title/Abstract] OR "Cognitive Impairment" [Title/Abstract] OR "Age-Related Memory Disorders" [Title/Abstract] OR "Mild Cognitive Impairment" [Title/Abstract] OR "MCI" [Title/Abstract] OR "Mild Neurocognitive Disorder" [Title/Abstract] OR"Cognitive Decline" [Title/Abstract] OR "Mental Deterioration" [Title/Abstract]

#3 #1 OR #2

#4 "Acupuncture therapy"[Mesh]

#5 "acupuncture therapy"[Title/Abstract] OR "acupuncture"[Title/Abstract] OR "acupuncture-moxibustion"[Title/Abstract] OR "meridian*"[Title/Abstract] OR "acupoint*"[Title/Abstract] OR "warm needling"[Title/Abstract] OR "warm acupuncture"[Title/Abstract] OR "acupuncture plus moxibustion"[Title/Abstract] OR "electronic acupuncture"[Title/Abstract] OR "electro-acupuncture"[Title/Abstract] OR "electroacupuncture"[Title/Abstract] OR "fire acupuncture"[Title/Abstract] OR "auricular needle"[Title/Abstract] OR "scalp needle"[Title/Abstract] OR "abdominal needle"[Title/Abstract] OR "wrist ankle needle"[Title/Abstract] OR "triple puncture"[Title/Abstract] OR "dry needle"[Title/Abstract] OR "needle"[Title/Abstract] OR "body acupuncture"[Title/Abstract] OR "manual-acupuncture"[Title/Abstract]

#6 #4 OR #5

#7 "neuroimaging"[MeSH] OR "magnetic resonance imaging"[Mesh] OR "Positron-Emission Tomography"[Mesh] OR "magnetic resonance imaging"[Mesh]

#8 "neuroimaging"[Title/Abstract] AND "magnetic resonance imaging"[Title/Abstract] OR "Positron-Emission Tomography"[Title/Abstract] OR "functional magnetic resonance imaging"[Title/Abstract] OR "fMRI"[Title/Abstract] OR "functional image"[Title/Abstract] OR "Diffusion Tensor Imaging"[Title/Abstract] OR "DTI"[Title/Abstract] OR "structural magnetic resonance imaging"[Title/Abstract] OR "sMRI"[Title/Abstract] OR "functional near-infrared spectroscopy"[Title/Abstract] OR "electroencephalography"[Title/Abstract] OR "magnetic resonance spectroscopy"[Title/Abstract] OR "magnetoencephalography"[Title/Abstract]

#9 #7 OR #8

#10 #3 AND #6 AND #9

**EMBASE**

#1 'cognitive dysfunction'/exp/mj

#2 'cognitive dysfunction':ti,ab,kw OR 'cognitive impairment':ti,ab,kw OR 'age-related memory disorders':ti,ab,kw OR 'mild cognitive impairment':ti,ab,kw OR mci:ti,ab,kw OR 'mild neurocognitive disorder':ti,ab,kw OR 'cognitive decline':ti,ab,kw OR 'mental deterioration':ti,ab,kw

#3 #1 OR #2

#4 'acupuncture therapy'/exp/mj

#5 'acupuncture therapy':ti,ab,kw OR acupuncture:ti,ab,kw OR 'acupuncture moxibustion':ti,ab,kw OR meridian*:ti,ab,kw OR acupoint*:ti,ab,kw OR 'warm needling':ti,ab,kw OR 'warm acupuncture':ti,ab,kw OR 'acupuncture plus moxibustion':ti,ab,kw OR 'electronic acupuncture':ti,ab,kw OR 'electro acupuncture':ti,ab,kw OR electroacupuncture:ti,ab,kw OR 'fire acupuncture':ti,ab,kw OR 'auricular needle':ti,ab,kw OR 'scalp needle':ti,ab,kw OR 'abdominal needle':ti,ab,kw OR 'wrist ankle needle':ti,ab,kw OR 'triple puncture':ti,ab,kw OR 'dry needle':ti,ab,kw OR needle:ti,ab,kw OR 'body acupuncture':ti,ab,kw OR 'manual acupuncture':ti,ab,kw

#6 #4 OR #5

#7 'neuroimaging'/exp OR 'magnetic resonance imaging'/exp OR 'Positron-Emission Tomography'/exp OR 'magnetic resonance imaging'/exp

#8 neuroimaging:ti,ab,kw OR magnetic resonance imaging:ti,ab,kw OR Positron-Emission Tomography:ti,ab,kw OR functional magnetic resonance imaging:ti,ab,kw OR fMRI:ti,ab,kw OR functional image:ti,ab,kw OR Diffusion Tensor Imaging:ti,ab,kw OR DTI:ti,ab,kw OR structural magnetic resonance imaging:ti,ab,kw OR sMRI:ti,ab,kw OR functional near-infrared spectroscopy:ti,ab,kw OR electroencephalography:ti,ab,kw OR magnetic resonance spectroscopy:ti,ab,kw OR magnetoencephalography:ti,ab,kw

#9 #7 OR #8

#10 #3 AND #6 AND #9

**Web of science**

TS=(‘cognitive dysfunction’ OR ‘cognitive impairment’ OR ‘age-related memory disorders’ OR ‘mild cognitive impairment’ OR ‘mci’ OR ‘mild neurocognitive disorder’ OR ‘cognitive decline’ OR ‘mental deterioration’) AND (‘acupuncture-moxibustion’ OR ‘meridian*’ OR ‘acupoint*’ OR ‘warm needling’ OR ‘warm acupuncture’ OR ‘acupuncture plus moxibustion’ ) AND (‘neuroimaging’ OR ‘magnetic resonance imaging’ OR ‘Positron-Emission Tomography’ OR ‘functional magnetic resonance imaging’ OR ‘fMRI’ OR ‘functional image’ OR ‘Diffusion Tensor Imaging’ OR ‘DTI’ OR ‘structural magnetic resonance imaging’ OR ‘sMRI’ OR ‘functional near-infrared spectroscopy’ OR ‘electroencephalography’ OR ‘magnetic resonance spectroscopy’ OR ‘magnetoencephalography’)

**Cochrane Library**

#1 Mesh descriptor: [Cognitive Dysfunction]explode all trees

#2 cognitive dysfunction:ti,ab,kw OR cognitive impairment:ti,ab,kw OR age-related memory disorders:ti,ab,kw OR mild cognitive impairment:ti,ab,kw OR mci:ti,ab,kw OR mild neurocognitive disorder:ti,ab,kw OR cognitive decline:ti,ab,kw OR mental deterioration:ti,ab,kw

#3 #1 OR #2

#4 Mesh descriptor: [acupuncture therapy] explode all trees;

#5 acupuncture therapy:ti,ab,kw OR acupuncture:ti,ab,kw OR acupuncture moxibustion:ti,ab,kw OR meridian*:ti,ab,kw OR acupoint*:ti,ab,kw OR warm needling:ti,ab,kw OR warm acupuncture:ti,ab,kw OR acupuncture plus moxibustion:ti,ab,kw OR electronic acupuncture:ti,ab,kw OR electro acupuncture:ti,ab,kw OR electroacupuncture:ti,ab,kw OR fire acupuncture:ti,ab,kw OR auricular needle:ti,ab,kw OR scalp needle:ti,ab,kw OR abdominal needle:ti,ab,kw OR wrist ankle needle:ti,ab,kw OR triple puncture:ti,ab,kw OR dry needle:ti,ab,kw OR needle:ti,ab,kw OR body acupuncture:ti,ab,kw OR manual acupuncture:ti,ab,kw

#6 #4 OR #5

#7 Mesh descriptor: [neuroimaging] explode all trees

#8 Mesh descriptor: [magnetic resonance imaging] explode all trees

#9 Mesh descriptor: [Positron-Emission Tomography] explode all trees

#10 Mesh descriptor: [magnetic resonance imaging] explode all trees

#11 neuroimaging:ti,ab,kw OR magnetic resonance imaging:ti,ab,kw OR Positron-Emission Tomography:ti,ab,kw OR functional magnetic resonance imaging:ti,ab,kw OR fMRI:ti,ab,kw OR functional image:ti,ab,kw OR Diffusion Tensor Imaging:ti,ab,kw OR DTI:ti,ab,kw OR structural magnetic resonance imaging:ti,ab,kw OR sMRI:ti,ab,kw OR functional near-infrared spectroscopy:ti,ab,kw OR electroencephalography:ti,ab,kw OR magnetic resonance spectroscopy:ti,ab,kw OR magnetoencephalography:ti,ab,kw OR electroencephalogram:ti,ab,kw

#12 #7 OR #8 OR #9 OR #10 OR #11

#13 #3 AND #6 AND #12

**CNKI**

(TKA=(‘针刺’+‘针灸’+‘体针’+‘手针’+‘电针’+‘温针’+‘头皮针’+‘头针’+‘耳针’+‘穴位’+‘火针’+‘腹针’+‘浮针’+‘三棱针’+‘九针’+‘经络’+‘经皮电刺激’+‘眼针’+‘舌针’+‘腕踝针’+‘刃针’+‘针刀’+‘刺络’+‘干针’) OR SU=(‘针刺疗法' + '针刺' + '针灸疗法’)) AND (TKA=(‘轻度认知障碍’+‘轻度认知功能障碍’+‘轻度认知损害’+‘轻度认知损伤’+‘轻度神经认知障碍’+‘认知减退’+‘精神衰退’)) AND (TKA=(‘神经影像’+‘脑影像’+‘磁共振技术’+‘功能磁共振’+‘结构磁共振’+‘弥散张量成像’+‘PET’+‘近红外成像’+‘脑电图’+‘脑磁图’) OR SU=(‘神经影像’+‘脑影像’))

**WF**

(题名或关键词:(轻度认知障碍 OR 轻度认知功能障碍 OR 轻度认知损害 OR 轻度认知损伤 OR 轻度神经认知障碍)) and (题名或关键词:(针刺 OR 针灸 OR 体针 OR 手针 OR 电针 OR 温针 OR 头皮针 OR 头针 OR 耳针 OR 穴位 OR 火针 OR 腹针 OR 浮针 OR 三棱针 OR 九针 OR 经络 OR 经皮电刺激 OR 眼针 OR 舌针 OR 腕踝针 OR 刃针 OR 针刀 OR 刺络 OR 干针) OR 主题:(针刺疗法 OR 针灸疗法 OR 针刺)) and (题名或关键词:(神经影像 OR 脑影像 OR 磁共振技术 OR 功能磁共振 OR 结构磁共振 OR 弥散张量成像 OR PET OR 近红外成像 OR 脑电图 OR 脑磁图) OR 主题:(神经影像 OR 脑影像))

**Chongqing VIP**

M=(轻度认知障碍 OR 轻度认知功能障碍 OR 轻度认知损害 OR 轻度认知损伤 OR 轻度神经认知障碍 OR 认知减退 OR 精神衰退) and M=(针刺 OR 针灸 OR 体针 OR 手针 OR 电针 OR 温针 OR 头皮针 OR 头针 OR 耳针 OR 穴位 OR 火针 OR 腹针 OR 浮针 OR 三棱针 OR 九针 OR 经络 OR 经皮电刺激 OR 眼针 OR 舌针 OR 腕踝针 OR 刃针 OR 针刀 OR 刺络 OR 干针) and R=(神经影像 OR 脑影像 OR 磁共振技术 OR 功能磁共振 OR 结构磁共振 OR 弥散张量成像 OR PET OR 近红外成像 OR 脑电图 OR 脑磁图)

**CBM**

1 "认知障碍" [加权:扩展]

2 "轻度认知障碍"[常用字段:智能] OR "轻度认知功能障碍"[常用字段:智能] OR "轻度认知损害"[常用字段:智能] OR "轻度认知损伤"[常用字段:智能] OR "轻度神经认知障碍"[常用字段:智能] OR "认知减退"[常用字段:智能] OR "精神衰退"[常用字段:智能]

3 1 OR 2

4 "针刺疗法"[加权:扩展] OR "针刺"[加权:扩展] OR "针灸疗法"[加权:扩展]

5 "针刺"[常用字段:智能] OR "针灸"[常用字段:智能] OR "体针"[常用字段:智能] OR "手针"[常用字段:智能] OR "电针"[常用字段:智能] OR "温针"[常用字段:智能] OR "头皮针"[常用字段:智能] OR "头针"[常用字段:智能] OR "耳针"[常用字段:智能] OR "穴位"[常用字段:智能] OR "火针"[常用字段:智能] OR "腹针"[常用字段:智能] OR "浮针"[常用字段:智能] OR "经络"[常用字段:智能] OR "经皮穴位电刺激"[常用字段:智能] OR "干针"[常用字段:智能]

6 4 OR 5

7 "神经影像" [加权:扩展] OR "脑影像"[加权:扩展]

8 "神经影像"[常用字段:智能] OR "脑影像"[常用字段:智能] OR "磁共振技术"[常用字段:智能] OR "功能磁共振"[常用字段:智能] OR "结构磁共振"[常用字段:智能] OR "弥散张量成像"[常用字段:智能] OR "PET"[常用字段:智能] OR "近红外成像"[常用字段:智能] OR "脑电图"[常用字段:智能] OR "脑磁图"[常用字段:智能]

9 7 OR 8

10 3 AND 4 AND 9
