# Supplementary material for: Acupuncture on mild cognitive impairment: A systematic review of neuroimaging studies
Source: Front Aging Neurosci. 2023 Feb 15;15:1007436. doi: 10.3389/fnagi.2023.1007436 (PMC9975578; doi:10.3389/fnagi.2023.1007436)
Supplement: Supplementary file 2 [file Table_2.DOCX]

**Appendix 2. Full-text articles excluded with reasons.**

| Full-text articles excluded | Reasons |
| --- | --- |
| Zhou et al.,2008 | Not neuroimaging study |
| Fan et al.,2020 | Not neuroimaging study |
| Zhang et al.,2021 | Not neuroimaging study |
| Yu et al.,2019 | Not neuroimaging study |
| Joo et al.,2018 | Ineligible intervention |
| Liu et al.,2022 | Ineligible intervention |
| Haiyan et al.,2021 | Ineligible intervention |

**References:**

Fan, D. Q., Zhao, H. C., Sheng, J., Liu, Y. R., Yu, J. (2020). Electroacupuncture modulates Resting-State functional connectivity in the default mode network for healthy older adults. *J Geriatr Psychiatry Neurol*. 33(2), 85-92. doi: 10.1177/0891988719868304

Haiyan, W., Siyu, L., Qiong, H., Haiyang, Y., Hong, Z. (2021). Effect on moxibustion on memory function and related serum protein markers in patients with amnestic mild cognitive impairment. *Acupuncture Research*. 46(09), 794-799

Joo, C. M., Kwen, K. N., Kang, H., 이고은, 이성익, and 김진원. (2018). Retrospective Analysis of Patients Suffering from Dementia or Mild Cognitive Impairment Treated by Collaboration between Western and Korean Medicine. *The Korean Society of Oriental Neuropsychiatry*. 29(2), 111-119

Liu, C., Zhao, L., Xu, K., Wei, Y., Mai, W., and Liang, L., et al. (2022). Altered functional connectivity density in mild cognitive impairment with moxibustion treatment: A resting-state fMRI study. *Brain Res.* 1775, 147732. doi: 10.1016/j.brainres.2021.147732

Yu, C. C., Ma, C. Y., Wang, H., Kong, L. H., Zhao, Y., and Shen, F., et al. (2019). Effects of Acupuncture on Alzheimer's Disease: Evidence from Neuroimaging Studies. *Chin. J. Integr. Med.* 25(8), 631-640. doi: 10.1007/s11655-018-2993-3

Zhang, J., Kuang, X., Tang, C.tive ability and brain fMRI after acupoint thread embedding in Alzheimer's disease patients]. *Zhongguo Zhong Xi Yi Jie He Za Zhi*. 28(8), 689-693

Zhou, Y. L., Han, H. Y., Jia, J. P. (2008). [Correlation analysis on changes between cognitive ability and brain fMRI after acupoint thread embedding in Alzheimer's disease patients]. *Zhongguo Zhong Xi Yi Jie He Za Zhi*. 28(8), 689-693
