# Supplementary material for: Acupuncture on mild cognitive impairment: A systematic review of neuroimaging studies
Source: Front Aging Neurosci. 2023 Feb 15;15:1007436. doi: 10.3389/fnagi.2023.1007436 (PMC9975578; doi:10.3389/fnagi.2023.1007436)
Supplement: Supplementary file 3 [file Table_3.docx]

**Appendix 3. Methodological quality assessments of randomised studies of the effects of interventions using RoB 2.**

| Study | Randomisation process | Deviations from intended interventions | Missing outcomes data | Measurement of the outcome | Selection of the reported results | Overall |
| --- | --- | --- | --- | --- | --- | --- |
| Li 2020 | Some concerns | Some concerns | Low | Low | Some concerns | Some concerns |
| Shan 2018 | Some concerns | Low | Low | Low | Some concerns | Some concerns |
| Xu 2017 | Some concerns | Some concerns | Low | Low | Some concerns | Some concerns |
| Tan 2017 | Some concerns | Low | Low | Low | Some concerns | Some concerns |
| Xu 2013 | Some concerns | Low | Low | Low | Some concerns | Some concerns |
| Jiang 2012 | Some concerns | Low | Low | Low | Some concerns | Some concerns |
| Cui 2011 | Some concerns | Low | Low | Low | Some concerns | Some concerns |
| Liu 2010 | Some concerns | Some concerns | Some concerns | Low | Some concerns | Some concerns |
| Jin 2010 | Some concerns | Some concerns | Low | Low | Some concerns | Some concerns |
| Liu 2009 | Some concerns | Some concerns | Low | Low | Some concerns | Some concerns |
