# Supplementary material for: Acupuncture on mild cognitive impairment: A systematic review of neuroimaging studies
Source: Front Aging Neurosci. 2023 Feb 15;15:1007436. doi: 10.3389/fnagi.2023.1007436 (PMC9975578; doi:10.3389/fnagi.2023.1007436)
Supplement: Supplementary file 4 [file Table_4.docx]

**Appendix 4. Methodological quality assessments of non-randomised studies of the effects of interventions using ROBINS-I.**

| Study | Bias due to confounding | Bias in selection of participants into study | Bias in classification of interventions | Bias due to deviations from intended interventions | Bias due to missing data | Bias in measurement of outcomes | Bias in selection of the reported result | Overall |
| --- | --- | --- | --- | --- | --- | --- | --- | --- |
| Khan 2022 | Moderate | Low | Low | Low | Low | Low | Low | Moderate |
| Cao 2021 | Moderate | Low | Low | Low | Low | Low | Low | Moderate |
| Wang 2020 | Moderate | Low | Low | Low | Low | Low | Low | Moderate |
| Ghafoor 2019 | Low | Low | Low | Low | Low | Low | Low | Low |
| Jia 2015 | Moderate | Low | Low | Low | Low | Low | Low | Moderate |
| Liu 2014 | Low | Low | Low | Low | Low | Low | Low | Low |
| Chen 2014 | Low | Low | Low | Low | Low | Low | Low | Low |
| Xu et al. 2013 | Low | Low | Low | Low | Low | Low | Low | Low |
| Chen 2013 | Low | Low | Low | Low | Low | Low | Low | Low |
| Wang 2012 | Low | Low | Low | Low | Low | Low | Low | Low |
| Feng 2012 | Moderate | Low | Low | Low | Low | Low | Low | Moderate |
| Jiang 2011 | Low | Low | Low | Low | Low | Low | Low | Low |
