# Supplementary material for: Acupuncture on mild cognitive impairment: A systematic review of neuroimaging studies
Source: Front Aging Neurosci. 2023 Feb 15;15:1007436. doi: 10.3389/fnagi.2023.1007436 (PMC9975578; doi:10.3389/fnagi.2023.1007436)
Supplement: Supplementary file 5 [file Table_5.DOCX]

**Appendix 5. Neuroimaging results after constant acupuncture.**

| **Study** | **Neuroimaging technologies** | **Neuroimaging results** |
| --- | --- | --- |
| Khan 2022 | fNIRS | MCI patients before needling vs. HC: *decreased hemodynamic response function* of the MCI in prefrontal cortex.  MCI patients before needling vs. MCI patients after needling: *increased* *hemodynamic response function* of the MCI patients obtained after constant acupuncture in prefrontal cortex. |
| Ghafoor 2019 | fNIRS | MCI patients before needling vs. HC: *decreased hemodynamic response function* of the MCI in prefrontal cortex.  MCI patients before needling vs. MCI patients after needling: *increased* hemodynamic response function, t-maps, FC of the MCI patients obtained after constant acupuncture in prefrontal cortex. |
| Liu 2010 | MRS | MCI patients before needling vs. MCI patients after needling /  AG vs. CM: *increased MI/Cr* and *Cho/Cr* in left temporal lobe. |
| Jin 2010 | MRS | MCI patients before needling vs. MCI patients after needling /  AG vs. CM: *increased MI/Cr* and *NAA/Cr* in left temporal lobe / hippocampus. |
| Liu 2009 | MRS | AG vs. CM: *increased MI/Cr* in left temporal tip and *NAA/Cr* in left hippocampus. |
| Xu 2017 | DTI (FA) | AG after needling vs. AG before needling: *increased FA values* in the knee of corpus callosum, splenium of corpus callosum, cingulate gyrus, inferior fronto-occipital fasciculus, superior longitudinal fasciculus |
|  | fMRI (ReHo) | AG after needling vs. AG before needling: *increased ALFF values* in the left temporal lobe, left parahippocampal gyrus, occipital lobe, lingual gyrus, precuneus. |
| Wang 2020 | fMRI (ALFF) | AG after needling vs. AG before needling: *increased ALFF values* in the right parahippocampal gyrus, left thalamus, right insula, left anterior cingulate gyrus and *decreased ALFF values* in the left posterior cerebellar lobe, bilateral inferior temporal gyrus, left inferior frontal gyrus, left middle temporal gyrus, left inferior occipital gyrus, left superior parietal lobule. |
| Li 2020 | fMRI (FC) | MCI patients before needling vs. HC: *increased* *FC* of the right HP and bilateral caudates; *decreased FC* of the right HP to the right inferior/middle temporal gyrus (ITG/MTG), the left amygdala, and the right fusiform.  AG after needling vs. AG before needling: *increased* *FC* of the the right hippocampal with right ITG/MTG, the right hippocampal FC with the right ITG and the left MTG. |
| Tan 2017 | fMRI (FC) | AG after needling vs. AG before needling: *increased* *FC* of the insula, dorsolateral prefrontal cortex, hippocampus, thalamus, inferior parietal lobule, and anterior cingulate cortex. |

Notes: MCI: mild cognitive impairment; HC: healthy control; AG, acupuncture group; SAG, sham acupuncture group; CM: conventional medicine; fNIRS: functional near - infrared spectroscopy; DTI: diffusion tensor imaging; MRS: magnetic resonance spectroscopy; NAA: N-acetylaspartate; Cr: Creatine; Cho: Cholines; MI: Myo-Inositol; FA: fractional anisotropy; fMRI: functional magnetic resonance imaging; ALFF: amplitude of low frequency fluctuations; ReHo: regional homogeneity; FC: functional connectivity; ITG: inferior temporal gyrus; MTG: middle temporal gyrus.
