# Supplementary material for: Acupuncture on mild cognitive impairment: A systematic review of neuroimaging studies
Source: Front Aging Neurosci. 2023 Feb 15;15:1007436. doi: 10.3389/fnagi.2023.1007436 (PMC9975578; doi:10.3389/fnagi.2023.1007436)
Supplement: Supplementary file 6 [file Table_6.docx]

**Appendix 6. Neuroimaging results after instant acupuncture.**

| **Study** | **Neuroimaging technologies** | **Neuroimaging results** |
| --- | --- | --- |
| Jia 2015 | fMRI (ALFF) | MCI patients before needling vs. HC: *increased* *ALFF* of the right inferior temporal gyrus and posterior cingulate cortex; *decreased ALFF* of the left medial frontal gyrus.  AG after needling vs. HC: *increased* *ALFF* of the postcentral gyrus, right medial frontal gyrus, cerebellar tonsil culmen, brainstem culmen, and left cuneus.  SAG after needling vs. HC: *decreased ALFF* of the left medial frontal gyrus and right superior frontal gyrus.  AG after needling vs. AG before needling: *increased ALFF* of the left parahippocampal gyrus, cingulate gyrus, middle frontal gyrus, right middle frontal gyrus, and subthalamic gyrus.  SAG after needling vs. SAG before needling: *increased* *ALFF* of the left precentral gyrus, both right and left medial frontal gyrus, and *decreased* *ALFF* of the right superior temporal gyrus.  AG after needling vs. SAG after needling: *increased* *ALFF* of right superior temporal gyrus, cingulate gyrus, and *decreased* *ALFF* of middle front gyrus. |
| Liu 2014 | fMRI (ReHo) | MCI patients before needling vs. HC: *increased* *Reho* of precentral gyrus, superior frontal gyrus, and insula, and *decreased* *Reho* of middle temporal gyrus, parahippocampal and cingulate cortex.  AG after needling vs. AG before needling: *increased Reho* of the precuneus, and cingulate cortex.  AG after needling vs. HC after needling: *increased Reho* of the middle temporal gyrus, superior parietal lobule, middle frontal gyrus, supramarginal, and precentral gyrus, and *decreased Reho* of the frontal regions, parahippocampal, and posterior cingulated cortex. |
| Xu et al. 2013 | fMRI (FC) | AG after needling vs. AG before needling: *increased FC* of the left dorsal lateral prefrontal cortex and bilateral frontal lobe, bilateral frontal lobes, left frontal and left dorsolateral prefrontal cortex and left anterior prefrontal cortex, and *decreased FC* of the bilateral inferior parietal lobule. |
| Feng 2012 | fMRI (FC) | MCI patients before needling vs. HC: *increased* *FC* of parahippocampal gyrus, thalamus, and *decreased* *FC* of the temporal regions (hippocampus, thalamus,  fusiform gyrus) and the prefrontal cortex (inferior frontal gyrus, orbital, olfactory cortex, superior frontal gyrus, middle frontal gyrus).  AG after needling vs. AG before needling: *increased* *FC* of hippocampus, olfactory cortex, superior frontal gyrus, insula, parahippocampal gyrus and cingulate cortex. |
| Chen 2013 | fMRI (EC) | Dorsolateral prefrontal cortex and hippocampus emerged as central hubs |
| Cao 2021 | fMRI (CNA) | AG during needling vs. AG before needling: *positive activation areas* of left inferior frontal gyrus, right inferior frontal gyrus, inferior frontal gyrus, right insular, left superior temporal gyrus, right inferior temporal gyrus, right anterior cerebellar lobe, posterior lobe and right thalamus; *negative activation areas* of left posterior cingulate gyrus, left precuneus, and right hippocampal gyrus. |
| Chen 2014 | fMRI (CNA) | AG during needling vs. AG before needling: *positive activation areas* of bilateral anterior cingulate gyrus, left medial frontal cortex, left cuneus, left middle frontal gyrus, left lingual gyrus, right medial frontal gyrus, bilateral inferior frontal gyrus, left superior frontal gyrus, right cuneus, right superior temporal gyrus, left subcallosal gyrus, bilateral precuneus, right medial frontal gyrus, right superior frontal, left cingulate gyrus, left precentral gyrus, and right fusiform gyrus. |
| Xu 2013 | fMRI (CNA) | AG during needling vs. AG before needling: *positive activation areas* of bilateral medial frontal gyrus, left cerebellar vermis, left posterior central gyrus, left insula |
| Jiang 2012 | fMRI (CNA) | AG after needling vs. AG before needling: *positive activation areas* of right cingulate gyrus, bilateral medial frontal gyrus, left posterior central gyrus |
| Wang 2012 | fMRI (CNA) | MCI patients before needling vs. HC: *positive activation areas* of the temporal lobe [left middle temporal gyrus], frontal lobe [left superior frontal gyrus, left middle frontal gyrus and bilateral inferior frontal gyrus] and left lentiform nucleus; *negative activation areas* of the right cingulate gyrus and left fusiform gyrus.  AG during needling vs. AG before needling: *positive activation areas* of the bilateral cerebellum posterior lobe, temporal lobe [bilateral middle temporal gyrus, bilateral fusiform gyrus, right parahippocampus, left inferior temporal gyrus], frontal lobe, parietal lobe [bilateral inferior parietal lobule and right postcentral gyrus] and occipital lobe; *negative activation areas* of the bilateral cerebellum posterior lobe, temporal lobe, frontal lobe [(bilateral superior frontal gyrus, right middle frontal gyrus, left precentral gyrus)], parietal lobe [(right postcentral gyrus, left paracentral lobule, left superior parietal lobule, right lingual gyrus and limbic regions.  AG after needling vs. AG before needling: *positive activation areas* bilateral cerebellum posterior lobe, temporal lobe (bilateral fusiform gyrus, right middle temporal gyrus and right parahippocampus), frontal lobe, right lentiform nucleus, left extra nuclear and right thalamus; *negative activation areas* of the bilateral cerebellum posterior lobe, temporal lobe (bilateral middle temporal gyrus, left superior temporal gyrus, right inferior temporal gyrus, and right fusiform gyrus), frontal lobe (left superior frontal gyrus, left inferior frontal gyrus, bilateral precentral gyrus, right middle frontal gyrus), parietal lobe (bilateral postcentral gyrus, left inferior parietal lobule, bilateral superior parietal lobule, right angular) and occipital lobe [left superior occipital lobe, left cuneus] |
| Jiang 2011 | fMRI (CNA) | AG during needling vs. AG before needling: *positive activation areas* of the bilateral medial frontal gyrus, left posterior central parietal gyrus, left insula, right superior temporal gyrus, left cerebellar vermis; *negative activation areas* of the left superior frontal gyrus, cingulate cortex, right superior temporal gyrus |
| Cui 2011 | fMRI (CNA) | MCI patients before needling vs. HC: *negative activation areas* of the right superior temporal gyrus, left cingulate gyrus and left superior frontal gyrus .  AG during needling vs. AG before needling: *positive activation areas* of the bilateral medial frontal gyrus, left cerebellar vermis, left posterior central gyrus, left insula, left superior temporal gyrus. |
| Shan 2018 | fMRI (CNA) | AG during needling vs. AG before needling: *positive activation areas* of the left supramarginal gyrus, left superior temporal gyrus, left rolandic operculum, left cerebellum, right middle frontal gyrus, and right inferior frontal gyrus; *negative activation area* of the lef inferior parietal gyrus. |

Notes: MCI: mild cognitive impairment; HC: healthy control; AG, acupuncture group; SAG, sham acupuncture group; fMRI: functional magnetic resonance imaging; ALFF: amplitude of low frequency fluctuations; ReHo: regional homogeneity; FC: functional connectivity; EC: effective connectivity; CNA: cerebral neurons activation.
